# The ADDITION-Cambridge trial protocol: a cluster – randomised controlled trial of screening for type 2 diabetes and intensive treatment for screen-detected patients

**DOI:** 10.1186/1471-2458-9-136

**Published:** 2009-05-12

**Authors:** Justin B Echouffo-Tcheugui, Rebecca K Simmons, Kate M Williams, Roslyn S Barling, A Toby Prevost, Ann Louise Kinmonth, Nicholas J Wareham, Simon J Griffin

**Affiliations:** 1 MRC Epidemiology Unit, Box 285, Addenbrooke's Hospital, Hills Road, Cambridge, CB2 0QQ, UK; 2 General Practice and Primary Care Research Unit, Department of Public Health and Primary Care, University of Cambridge, Cambridge, CB2 0SR, UK

## Abstract

**Background:**

The increasing prevalence of type 2 diabetes poses a major public health challenge. Population-based screening and early treatment for type 2 diabetes could reduce this growing burden. However, the benefits of such a strategy remain uncertain.

**Methods and design:**

The *ADDITION-Cambridge *study aims to evaluate the effectiveness and cost-effectiveness of (i) a stepwise screening strategy for type 2 diabetes; and (ii) intensive multifactorial treatment for people with screen-detected diabetes in primary care. 63 practices in the East Anglia region participated. Three undertook the pilot study, 33 were allocated to three groups: no screening (control), screening followed by intensive treatment (IT) and screening plus routine care (RC) in an unbalanced (1:3:3) randomisation. The remaining 27 practices were randomly allocated to IT and RC. A risk score incorporating routine practice data was used to identify people aged 40–69 years at high-risk of undiagnosed diabetes. In the screening practices, high-risk individuals were invited to take part in a stepwise screening programme. In the IT group, diabetes treatment is optimised through guidelines, target-led multifactorial treatment, audit, feedback, and academic detailing for practice teams, alongside provision of educational materials for newly diagnosed participants. Primary endpoints are modelled cardiovascular risk at one year, and cardiovascular mortality and morbidity at five years after diagnosis of diabetes. Secondary endpoints include all-cause mortality, development of renal and visual impairment, peripheral neuropathy, health service costs, self-reported quality of life, functional status and health utility. Impact of the screening programme at the population level is also assessed through measures of mortality, cardiovascular morbidity, health status and health service use among high-risk individuals.

**Discussion:**

*ADDITION-Cambridge *is conducted in a defined high-risk group accessible through primary care. It addresses the feasibility of population-based screening for diabetes, as well as the benefits and costs of screening and intensive multifactorial treatment early in the disease trajectory. The intensive treatment algorithm is based on evidence from studies including individuals with clinically diagnosed diabetes and the education materials are informed by psychological theory. *ADDITION-Cambridge *will provide timely evidence concerning the benefits of early intensive treatment and will inform policy decisions concerning screening for type 2 diabetes.

**Trial registration:**

Current Controlled trials ISRCTN86769081

## Background

Diabetes is an increasingly common problem [1], associated with a substantial burden of premature mortality, morbidity, suffering and financial cost through its macrovascular and microvascular complications [2]. The high proportion (30–50%) of undiagnosed cases of diabetes [3], the large number of individuals with complications at clinical diagnosis [4], and the long (9–12 years) latent phase of the condition [5]. Indeed, type 2 diabetes fulfils many of the criteria for suitability for screening [6]. Adopting a national policy of population-based screening for type 2 diabetes could help to reduce the current burden of morbidity and mortality associated with the disease. However, there is continuing uncertainty about the benefits and costs of screening for type 2 diabetes. In particular, modelling data suggest that a key but uncertain determinant of the cost-effectiveness of screening is the size of cardiovascular risk reduction consequent on early intensive multifactorial treatment in screen-detected patients [7]. There is evidence that intensive multifactorial treatment is cost-effective in reducing cardiovascular morbidity and mortality in patients further along the disease trajectory with microalbuminuria [8,9]. It is also clear that intensive treatment of individual cardiovascular risk factors (blood pressure and cholesterol) is beneficial [10-14]. However, it is unclear to what extent intensive multifactorial treatment among screen-detected patients would be cost-effective. Intensive treatment of hyperglycaemia among patients with long-standing diabetes has not been associated with cardiovascular benefits [15-17]. However, long term follow-up of the UKPDS cohort showed that reducing levels of blood glucose from diagnosis led to fewer cardiovascular events [18]. It is unclear whether intensive treatment of hyperglycaemia during the lead time between clinical diagnosis and diagnosis by screening will be associated with similar benefits.

Ideally, there should be trial evidence of cost-effectiveness of screening programmes before they become public policy [6], as was the case for ultrasonographic screening for abdominal aortic aneurysm in men [19]. This is not yet the case for type 2 diabetes. TheAnglo-Danish-Dutch Study of Intensive Treatment In People with Screen Detected Diabetes in Primary Care(*ADDITION) *trial was set up in three countries: England (Cambridge and Leicester), Denmark and The Netherlands to provide evidence on screening for type 2 diabetes and the effects of early intensive multifactorial treatment [20]. We present the protocol of the Cambridge component of this trial.

### Target population

If population-based screening for type 2 diabetes were to be undertaken, current evidence supports a targeted approach [6]. The *ADDITION-Cambridge *study targets people without known diabetes but at high risk of having prevalent undiagnosed type 2 diabetes, identified using a previously validated risk score [21]. This risk tool combines information routinely collected in primary care, including age, sex, body mass index and prescribed medication (steroids and antihypertensive drugs), to predict the presence of undiagnosed diabetes. This simple practical tool has previously been shown to perform reasonably well in different settings[22,23].

### Limited evidence from previous studies

#### (i) The potential benefits and harms of screening

Earlier detection of diabetes and treatment of hyperglycaemia and related metabolic abnormalities may be beneficial. Screening for hyperglycaemia can identify patients at an early stage of the disease [24,25] who are likely to benefit from intensive treatment of cardiovascular risk factors. Patients who are given the label of diabetes may also benefit from becoming involved in a more organised and effective system of risk factor management [26]. However, it is uncertain whether an intervention to promote intensive multifactorial management for patients with screen-detected diabetes in primary care will be cost-effective. It is also unclear whether such an intervention might impact on the care of other patients with established diabetes and those at risk of diabetes in the primary care practices undertaking intensive treatment.

Concerns have been expressed about the psychological harms of screening programmes [27]. With the exception of one small randomised trial undertaken in the pilot phase of *ADDITION-Cambridge *[28], published data suggest no or limited psychological impact of screening for diabetes in newly detected individuals [29]. These data, which were mainly derived from cross-sectional or cohort studies (susceptible to selection and ascertainment bias) were recently confirmed by the results of a controlled trial embedded in *ADDITION-Cambridge *[30]. However, none of the published studies have examined the wider impact of screening on health related quality of life among high-risk groups, the potential for a worsening of risk due to false reassurance, or the subsequent effects of intensive treatment on the quality of life of screen-detected individuals.

Despite screening negative for diabetes, some of the high-risk people targeted in a screening programme will exhibit a high cardiovascular risk profile and/or develop diabetes within a relatively short period of time given their high lifetime risk compared to the general population [31]. Screening and promotion of early multifactorial intensive treatment could therefore have a wider impact among high-risk individuals as well as those diagnosed with diabetes as a result of screening.

Little is known about the impact at the population level on mortality of a screening programme for diabetes. Modelling studies have suggested that 4–5 yearly screening programmes might be associated with a significant reduction in diabetes-related mortality in the order of 26–40% [32,33]. However, this needs to be confirmed in formal prospective studies.

#### (ii) The lack of trial evidence

Evaluations of screening that do not incorporate random allocation of representative population samples are particularly susceptible to misinterpretation and overestimation of benefits due to lead time, length time, spectrum, ascertainment and selection bias [34]. Evidence from randomised trials of the impact of screening is important for public health policy decisions in view of the extensive organisational, technical and financial inputs such a screening programme would demand. There is no trial evidence to suggest that early detection of type 2 diabetes improves outcomes, or that treatment effective for clinically diagnosed patients produces greater benefit when commenced in the lead time between detection by screening and clinical diagnosis.

### ADDITION-Cambridge Objectives

The primary objective of the *ADDITION-Cambridge *study is to evaluate the effectiveness and cost-effectiveness of a stepwise screening programme for type 2 diabetes and intensive multifactorial treatment in people with screen-detected diabetes in English general practice.

The following research questions are posed:

• *Feasibility of screening*: What uptake and yield are achievable in a primary care-based stepwise screening programme for type 2 diabetes?

• *Costs of screening*: What are the health service and patient costs of screening for type 2 diabetes?

• *Early treatment of type 2 diabetes*: Can an optimised intensive intervention targeting blood glucose and associated cardiovascular risk factors reduce cardiovascular risk and mortality in people with screen-detected diabetes? Is this intervention cost-effective?

• *Population level impact: *Is a targeted screening programme for type 2 diabetes associated with reductions in population mortality and morbidity?

## Methods and design

### Design

**Figure 1:**
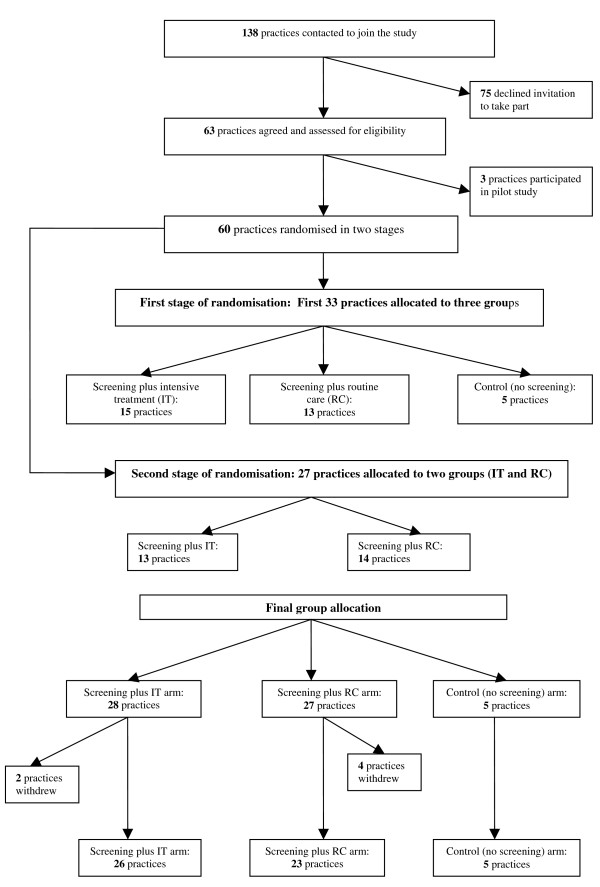
**Practice recruitment and randomisation in the *ADDITION-Cambridge *study**.

**Figure 2:**
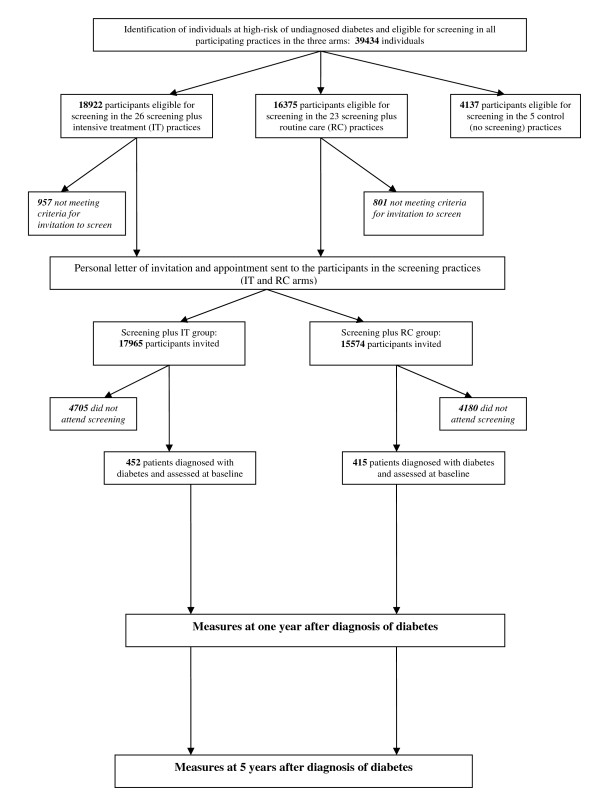
**Participant recruitment in the *ADDITION-Cambridge *study**.

*ADDITION-Cambridge *consists of two phases: a screening study and a subsequent treatment study. The screening phase examines the feasibility of a stepwise procedure to identify people with undetected diabetes and the effects of screening on health outcomes at the population level. The treatment study is a pragmatic single blind, cluster-randomised, parallel group trial comparing the effects of intensive multifactorial therapy with routine care (according to national guidelines) in individuals with screen-detected type 2 diabetes. The evaluation of the impact of the screening programme at the population level through the inclusion of random allocation of practices to a no screening (control) group is a feature specific to *ADDITION-Cambridge*. The study design, practice and patient flows are shown in Figures 1 and 2.

Ethical approval was obtained from the Cambridge (ref:01/063), Huntingdonshire (ref:00/609), Peterborough and Fenland (ref:P01/95), West Essex (ref:1511-0103), North and Mid Essex (ref:MH395 MREC02/5/54), West Suffolk (ref:03/002), and Hertfordshire and Bedfordshire (ref:EC03623) Local Research Ethics Committees, and the Eastern Multi-Centre Research Ethics Committee (ref:02/5/54). Written informed consent was obtained for all participants involved in both phases of the *ADDITION-Cambridge *study at the time of the diabetes screening appointment and subsequent diagnostic test.

*ADDITION-Cambridge *is registered as ISRCTN86769081. The ClinicalTrials.gov Identifier of the whole *ADDITION *Study that includes England (Cambridge and Leicester), Denmark and the Netherlands is NCT00237549.

### Setting

Patients were recruited from general practices in urban, suburban and rural Cambridgeshire, East Hertfordshire, West Suffolk and North Essex areas of England.

### Practice recruitment

Figure 1 shows the flow of practice recruitment. 138 practices were invited to take part in the study between September 2001 and August 2003. Personalised letters were sent to the practice manager, partners and nursing staff in each surgery highlighting the importance of the study to primary care, the involvement of practice staff and the reimbursement of all costs involved. We enclosed a brief summary of the study and a Research Information Sheet for Practices [35]. A principal investigator and member of the trial team visited interested practice teams to discuss the study in further detail. All relevant practice staff were encouraged to attend, particularly those that would be involved in the administration of the screening programme. Practices were eligible if they were able to provide data for the calculation of the diabetes risk score for at least 70% of their patients, a criterion satisfied by all 63 practices that agreed to take part.

Three practices undertook pilot testing of the screening strategy, the baseline measures and the intensive treatment materials and training. The remainder (60 practices) were allocated to the three study arms. In the participating practices, a "set-up" visit was undertaken to deliver practice study manuals, to provide the software developed to assist with monitoring the progress of the screening programme and recording of blood glucose test results, and to train the staff in logistical and technical aspects of screening. Further visits were arranged for practices allocated to screening followed by intensive treatment to provide the materials and training to enable them to deliver the intervention.

### Practice randomisation

Randomisation of practices was completed centrally and independently of the trial co-ordination team immediately following recruitment. The project statistician used a partial minimisation procedure that dynamically adjusted the randomisation probabilities to balance important baseline practice variables: the number of patients with known diabetes (<160 and ≥ 160 patients) and the local district hospital (Addenbrooke's, Hinchingbrooke, Peterborough, Kings Lynn, Broomfield, Stevenage and Bury St Edmunds hospitals). The first 33 practices recruited were allocated in a ratio of 1:3:3 to the following arms: control (no screening), screening followed by intensive multi-factorial treatment of diabetes (IT), and screening plus routine care of diabetes according to national guidelines (RC). Allocation of practices to the control (no screening) group was stopped at N = 5. The need to increase the yield of individuals with diabetes for the treatment trial warranted the uneven randomisation ratio with a disproportionate number of screening practices and a second stage of randomisation. 27 practices were subsequently randomised in a ratio of 1:1 to IT (n = 13) and RC (n = 14). The final group allocation after the two stages of randomisation included 28 practices to IT, 27 practices to RC and 5 practices to control (no screening). Six of the 60 randomised practices (2 IT and 4 RC) dropped out following recruitment, but before screening commenced due to other commitments or unforeseen difficulties in setting up the practice-based screening programme.

### Phase one: step-wise screening programme

#### (i) Eligibility for screening

Individuals eligible for an invitation for screening were people registered with one of the participating general practices, aged 40 to 69 years, not known to have diabetes and with a diabetes risk score of >0.17 (corresponding to the top 25% of the population distribution). In screening practices, eligible participants deemed unfit for screening by their general practitioner were not invited for biochemical testing. Exclusion criteria, also assessed initially by the participating general practitioners, included pregnancy, lactation, an illness with a likely prognosis of less than one year or a psychiatric illness likely to limit study involvement or invalidate informed consent.

#### (ii) Participant recruitment

Figure 2 outlines the recruitment procedure. Participants were recruited through their local general practice. An electronic search of medical records was undertaken for routinely collected information that would allow the calculation of a diabetes risk score for each patient [21]. Information about the diabetes risk score was withheld from practitioners in the control practices.

**Figure 3:**
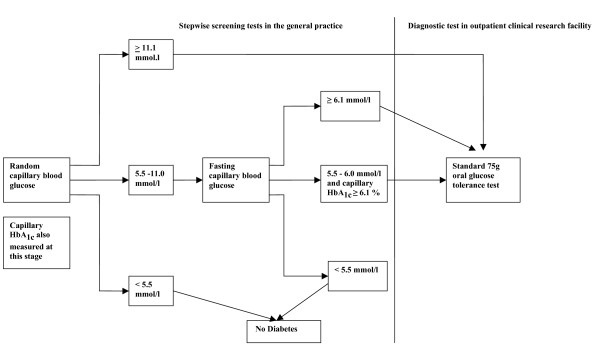
**Screening algorithm used in the *ADDITION-Cambridge *study**.

Figure 3 outlines the screening and diagnostic tests used to identify people with undiagnosed diabetes. In practices in the RC and IT groups, general practitioners wrote to all high-risk patients, enclosing a study information sheet, and inviting them to attend the practice for random capillary blood glucose (RBG) and capillary glycosylated haemoglobin (HbA_1c_) tests, after initial consent had been obtained. The letter was sent at least two weeks in advance of the scheduled appointment. Patients were advised to telephone the surgery and arrange an alternative appointment if the original was inconvenient. One reminder letter was sent to non-attendees. Participants with an RBG of ≥ 11.1 mmol/l were invited for a standard 75 g oral glucose tolerance test (OGTT) at one of four outpatient facilities. Those with an RBG of 5.5–11.0 mmol/l were invited to return to the practice for a fasting capillary blood glucose (FBG) test. Those with an FBG of ≥ 6.1 mmol/l, or an FBG of 5.5–6.0 mmol/l together with an HbA_1c _of ≥ 6.1%, were invited for an OGTT. The RBG, FBG and OGTT were conducted on different days. Participants with an FBG of 5.5–6.0 mmol/l and an HbA_1c _of ≥ 6.1% who had a positive OGTT underwent a second confirmatory OGTT on a different day. World Health Organisation criteria were used to diagnose diabetes [36]. Practitioners were informed by fax about the result of clinical and biochemical measures with a clear statement of whether or not the individual met diagnostic criteria for type 2 diabetes. The general practitioner or a practice nurse then informed the patient of the test results.

In the 54 participating practices (including the five control practices), 39,434 people aged 40–69 years were at high risk of prevalent undiagnosed diabetes. In the 49 screening practices, 35,297 individuals aged 40–69 years were at high-risk. 33,539 patients were invited for the first stage of screening (RBG and HbA_1c_) and 24,654 (73.5%) attended this appointment.

#### (iii) Outcomes

These include the number of high-risk individuals presenting for screening, the number of people with newly diagnosed type 2 diabetes, the psychological status of people invited for screening, metabolic status, cardiovascular risk and self-perceived health in people with newly-diagnosed type 2 diabetes, and health service and patient costs. In addition we will assess the population effects of the screening programme by comparing high-risk individuals in the three study groups (IT, RC and control) using the following measures: mortality, self-reported cardiovascular morbidity, health status, health utility and lifestyle changes (self-reported diet, physical activity and smoking status). Mortality will be assessed on all high-risk individuals, while other measures will be collected in a random sample of the high-risk population (in each of the three groups: IT, RC and control) using a postal questionnaire. All the high-risk participants in the three study arms are tagged at the Office of National Statistics (ONS) for mortality, following approval under section 60 of the UK Health and Social Care Act 2001 (Reference MR798).

### Phase two: trial of intensive multifactorial treatment in people with screen-detected diabetes

#### (i) Intervention

Participants are treated routinely or intensively depending on the study arm to which their practice was randomised (RC or IT). The intensification of diabetes management is achieved through the addition of the following features to the existing diabetes care within IT practices:

• Funding of practices to facilitate more frequent contact between patients and practitioners. The recommended frequency of consultation was one 30-minute annual review for each patient, three additional 10-minute consultations with a GP and three with a nurse, per year for the first three years after diagnosis, over and above the usual consultation frequency for a patient aged 40–69 years.

• Recommendation to the GPs to refer all newly diagnosed patients to a dietitian

• A practice-based academic detailing session for practitioners conducted by a local consultant diabetologist and a general practice opinion leader to describe the treatment algorithms and targets, patient materials, and present the evidence underpinning intensive treatment. The treatment algorithms (Table 1) were based on trial data demonstrating the benefits of intensive treatment of several cardiovascular risk factors in people with diabetes [8,13]. All treatment recommendations were for medications within their existing licensed indications. GPs were advised to consider prescribing an angiotensin converting enzyme (ACE) inhibitor to patients with a blood pressure ≥ 120/80 mmHg and a previous cardiovascular event or at least one cardiovascular risk factor other than diabetes [13]. The rest of the intervention is based on the stepwise regimen from the Steno-2 study [8] aimed at optimising hyperglycaemia, hypertension, dyslipidaemia and microalbuminuria. As per the Steno-2 regime, GPs were advised to consider prescribing 75 mg of aspirin daily to all patients without specific contraindications. Although targets for treatment are specified and classes of drugs recommended, where there is a choice of individual agents the decision is made by the GPs and patients. The intensive treatment protocol was reviewed after the publication of the Heart Protection Study [12] to include the prescription of statins to all screen-detected patients with a cholesterol level of ≥ 3.5 mmol/l.

**Table 1:** Treatment recommendations in the intensive treatment arm

	**Target**	**Baseline**	**2 months** **If above** **target**	**4 months** **If above ** **target**	**6 months** **If above ** **target**	**9 months** **If above** **Target**	**12 months** **If above ** **target**
HbA1c	**<7.0%**	**Diet**	HbA1c >6.5% **Metformin** (avoid using metformin if creatinine level >130 μmol/L)	HbA1c >6.5% add a second medication **Metformin ** **or PGR or ** **SU or TZD**	HbA1c >6.5% add a third medication **Metformin ** **or PGR or** **SU or TZD**	Continue oral hypoglycaemic medication and consider adding insulin	As for 9 months
Blood Pressure	≤ **135/85mmHg**	>120/80 mmHg or CVD+ **ACE Inhibitor ** titrated to maximum dose	If bp >135/85 mmHg Add a **Thiazide ** **diuretic or Ca** ** Antagonist** (Change **ACE to ACE2 ** if creatinine >130 μmol/L or K+ >5.0 mmol/L or intolerable side effects)	As for 2 months	If bp >135/85 mmHg **Add β blocker ** **or**α **Blocker**	As for 6 months	As for 6 months
Cholesterol †IHD-	**<5.0mmol/l**	Chol ≥ 3.5 mmol/l, **diet & statin**	Chol >5.0 mmol/l **Increase statin** ** dose up to ** **maximum** (If CK> 1800 U/L, stop statin)	As for 2 months	Consider adding a **fibrate** if Chol >5.0 mmol/l	As for 6 months	As for 6 months
Cholesterol IHD+	**<4.5mmol/l**	chol ≥ 3.5 mmol/, **diet & statin**	Chol >4.5 mmol/l **Increase statin ** **dose up to** ** maximum** (If CK> 1800 U/L, stop statin)	As for 2 months	Consider adding a **fibrate **if Chol >5.0 mmol/l	As for 6 months	As for 6 months
Acetylsalycilic acid	75 mg of aspirin daily to all patients without specific contraindications						

SU = Sulphonylurea, PGR = Prandial glucose regulator, ACE = angiotensin converting enzyme, TZD = thiazolinedione, ACE2: Angiotensin- II receptor Antagonist, K+: potassium, Ca: Calcium, IHD- = no history of ischaemic heart disease, IHD+ = history of ischaemic heart disease, CVD+ = Previous cardiovascular event or presence of cardiovascular risk factor other than diabetes, bp = blood pressure, Chol = cholesterol

• Two interactive practice-based audit and feedback sessions were undertaken, including feedback of the overall performance of the practice against the treatment targets, the optimisation of the management of individual patients and the reiteration of the treatment targets. These were organised by the same opinion leaders at six and 14 months after the initial education session.

• Provision of glucometers for patients and any necessary training in their use for practitioners. The decision to offer a glucometer or not to a patient was left to the practitioner.

• Practice teams were provided with a pack of theory-based educational materials (Getting Started with Diabetes) to give to patients at diagnosis. The materials provide a shared framework on the causes, consequences and treatment of diabetes. The materials were developed by a multidisciplinary team and drew on Leventhal's self regulation model, a social cognition model from psychology [37]. They cross-referred to 'Diabetes for Beginners-Type 2' a Diabetes UK publication [38] that was included in the patient's pack. Specifically, participants were encouraged (i) to try to lose 5–10% of their body weight (relevant if BMI>28 kg/m^2 ^with a target of 0.45 kg/week) through a combination of diet and physical activity; (ii) to increase their physical activity gradually (the goal was to reach the equivalent of 35 minutes of brisk walking per day for 7 days per week); (iii) to avoid excessive alcohol intake; (iv) to take their medication regularly; (v) to self-monitor their blood glucose level if given a glucometer by their practice (the targets for self-monitored blood glucose are < 9 mmol/l 90 minutes after meals, and < 6 mmol/l before meals), and: (vi) to attend annual checks. Participants who smoked were encouraged to stop.

In the RC arm, participants with screen-detected diabetes receive the normal pattern of diabetes care as delivered through the UK National Health Service (NHS) according to current recommendations.

#### (ii) Endpoints

***Primary endpoints***: *At one year follow-up *the principle outcome is modelled 10-year risk of cardiovascular events derived using the UKPDS risk engine [39]. The UKPDS model uses information on sex, ethnicity, smoking status, presence or absence of atrial fibrillation, systolic blood pressure, HbA_1c_, total cholesterol, and HDL-cholesterol to predict the 10-year risk of primary CVD. Predicted events are myocardial infarction, sudden cardiac death, other incident ischaemic heart disease, stroke, and peripheral vascular disease death. *At five-year follow-up*, the primary endpoint is a composite of cardiovascular mortality and morbidity (non-fatal myocardial infarction, non-fatal stroke, non-traumatic amputations and revascularisations). ***Secondary endpoints ***are all-cause mortality, development or progression of renal impairment, peripheral neuropathy, blindness, reduced visual acuity, macular oedema, retinopathy; health status, health utility, quality of life, anxiety, well-being, treatment satisfaction, health service costs (number of visits to general practitioners and hospital doctors for outpatient clinics, hospital admissions and prescribed medications). ***Intermediate endpoints ***are self-reported smoking status, diet, physical activity behaviour and medication adherence, HbA_1c_, total cholesterol, LDL-cholesterol, HDL-cholesterol, triglycerides, blood pressure, modelled 10-year cardiovascular risk (at five-year follow-up), self reported hypoglycaemic episodes, microalbuminuria, body mass index and plasma vitamin C.

### Measurement

Table 2 shows the distribution across time of measures relating to the screening procedure and the treatment phase of the study. Baseline measurements were carried out on all patients eligible for an OGTT following the screening phase of the study. These included the completion of questionnaires, physiological and anthropometric measures and venesection. Similar measurements are conducted at one year and five years after diagnosis, without repetition of the OGTT. The measurements at baseline, one-year and five-year follow-up are undertaken at outpatient clinical research facilities by trained staff following standard operational procedures and unaware of participants' study group allocation. Questionnaires are used to collect information on basic demographics, health behaviours, health utility, functional status and costs.

**Table 2:** Measures used at baseline, one-and five-years in the *ADDITION-Cambridge*

Measures	**Baseline**			**1-year**			**5-year**		
	**C**	**RC**	**IT**	**C**	**RC**	**IT**	**C**	**RC**	**IT**
**Diabetes risk score calculation**	**X**	**X**	**X**						
**Questionnaires measures**									
1. Ethnic group, occupation, educational level and social class		**X**	**X**						
2 Smoking status, alcohol consumption		**X**	**X**		**X**	**X**	**X**	**X**	**X**
3. Rose angina questionnaire [51]		**X**	**X**				**X**		
4. Self-reported history of angina, heart attack and stroke		**X**	**X**		**X**	**X**	**X**	**X**	**X**
5. Medication adherence:									
All drugs during the last month [40]		**X**	**X**		**X**	**X**		**X**	**X**
Hypoglycaemic drugs during the last month [40]					**X**	**X**		**X**	**X**
6. EuroQoL EQ-5D [47] & SF-36 [45]/SF-8 [46]		**X**	**X**		**X**	**X**	**X**	**X**	**X**
7. Diabetes related quality of life: ADDQoL [44], Diabetes well-being: W-BQ 28 [44], Diabetes treatment satisfaction: DTSQ[44]					**X**	**X**		**X**	**X**
8. Spiegelberger Short form State Anxiety inventory [49]		**X**	**X**		**X**	**X**			
9. Consultation and relational empathy measure: CARE [48]		**X**	**X**		**X**	**X**			
10. Diabetes knowledge †					**X**	**X**			
11. EPAQ-2 [41]		**X**	**X**		**X**	**X**		**X**	**X**
12. IPAQ [42]		**X**	**X**		**X**	**X**	**X**	**X**	**X**
13. EPIC food frequency questionnaire [43]		**X**	**X**		**X**	**X**		**X**	**X**
14. Brief dietary questionnaire (adapted from the EPIC food frequency questionnaire) †							**X**	**X**	**X**
15. Costs comprising:									
Personal patient costs †		**X**	**X**						
Health Service and medication use previous 3 months (adapted from the Aberdeen Health Service Research Unit questionnaire) †					**X**	**X**	**X**	**X**	**X**
16. Neuropathy questionnaire (adapted from the Michigan Screening Instrument) †		**X**	**X**		**X**	**X**		**X**	**X**
**Biological measures**									
17. Waist circumference, height, weight, blood pressure, body fat impedance and ECG		**X**	**X**		**X**	**X**		**X**	**X**
18. Fasting capillary blood glucose		**X**	**X**						
19. Fasting, 30 and 120 min: venous whole blood glucose (OGTT), plasma glucose, plasma insulin.		**X**	**X**						
20. HbA_1c_, total cholesterol, HDL and LDL cholesterol, triglyceride, Vitamin C, Urinalysis, Urine albumin/creatinine ratio, Urea and Electrolytes, Creatinine, Albumin, Biliribin, Alanine Amino Transferanse (ALT), Alkaline Phosphatase, Aspartate Amino Transferase (AST), Thyroid Stimulating Hormone (TSH)		**X**	**X**		**X**	**X**		**X**	**X**
21. Modelled CVD risk calculated with the UKPDS risk engine [39]		**X**	**X**		**X**	**X**		**X**	**X**
22. Stereoscopic fundal photography								**X**	**X**
23. Mortality							**X**	**X**	**X**

†: Questionnaire developed for the study

#### Health behaviours

Smoking status, alcohol consumption, and medication adherence are assessed by questionnaire. Medication adherence is assessed by the Medication Adherence Report Schedule (MARS) questionnaire [40]. Physical activity is assessed using the EPAQ2 [41] and IPAQ [42] questionnaires. Dietary intake is evaluated using a validated food frequency questionnaire [43].

#### Health utility, functional status, quality of life, well-being, treatment satisfaction and anxiety

The generic and disease-specific instruments used are diabetes well-being questionnaire (W-BQ12) [44], SF-36 [45], SF-8 [46], Audit of Diabetes-Dependent Quality of Life (ADDQoL) [44], diabetes treatment satisfaction (DTS) [44], and EuroQol (EQ-5D) [47], consultation and relational empathy (CARE) measure [48] and the Spiegelberger Short form State Anxiety Inventory [49].

#### Costs

Personal patient costs to attend initial screening tests and health service use in the three months prior to follow-up are quantified using an adapted version of the Health Services Research Unit Aberdeen questionnaire that inquires about the use of services (consultations with healthcare professionals and hospitalisations) and medications [50].

Angina is assessed using the Rose angina questionnaire [51]. Neuropathy is evaluated using an adapted version of the Michigan Neuropathy Screening Instrument [52].

#### Physiological measures

Random and fasting capillary blood glucose concentrations were assessed by Hemocue (β-HemoCue AB, Angelholm, Sweden). The venous plasma blood glucose level is assessed by the glucose dehydrogenase method and read photochromatically. The stability of the analyser was checked daily and external calibration with the Hemocue quality assurance scheme was undertaken monthly. HbA_1c _was analysed in capillary blood samples from general practices using the Bio-Rad^® ^system and in venous samples at the time of diagnostic testing by ion-exchange high-performance liquid chromatography on a Tosoh machines (Tosoh Bioscience, Redditch, UK). Serum total cholesterol, HDL-cholesterol and triglycerides are measured by means of enzymatic techniques (Dade Behring Dimension analyser, Newark, USA). Plasma creatinine is analysed with kinetic colorimetric methods, urine albumin by rate nephelemetry (Dade Behring Nephelometer II, Newark, USA). Plasma levels of urea and electrolytes, bilirubin, alanine aminotansferase (ALT), aspartate aminotransferase (AST), alkaline phosphatase, and thyroid stimulating hormone (TSH) and urine levels of creatinine are assayed by means of the Dade Behring Dimension analyser. Plasma vitamin C level was measured with a Fluoroskan Ascent FL fluorometer. The albumin-to-creatinine ratio (ACR) is measured on a random spot urine specimen. For assays requiring fasting, participants attend after a 10-hour fast.

#### Anthropometry

Blood pressure is calculated as the mean of three measurements performed after at least 10 minutes rest, while participants are seated with the cuff on the predominant arm at the level of the heart, using an automatic sphygmomanometer (Omron M4, UK). ECG is recorded by a 12 lead machine. Body height and weight are measured in light indoor clothing and without shoes using a fixed rigid stadiometer and a scale (SECA, UK) respectively. Waist circumference is estimated as the average of two measurements taken with a tape measure halfway between the lowest point of the rib cage and the anterior superior iliac crests when standing. Body fat percentage is measured by bio-electrical impedance (TANITA, Tokyo, Japan).

#### Ascertainment of mortality and cardiovascular morbidity

Macrovascular and microvascular events in patients with screen-detected diabetes will be ascertained by a combination of strategies including electronic READ code searches of medical records for events, and notes extraction on potential cases of events. Anonymous case reports packs will be prepared by a member of the research team unaware of participants study group allocation for independent review of each potential event by an endpoint committee also unaware of study group allocation. All patients will also have an ophthalmologic evaluation including stereoscopic fundal photography at the five-year assessment. Fundal photography will be assessed by a separate endpoint committee blind to study groups. ICD-10 coded mortality data is reported periodically by the ONS for all high-risk participants in the three arms.

Assessment of the effect of screening in a random sample of people at high risk of prevalent undiagnosed diabetes in each of the three study groups (IT, RC and control) will be undertaken by postal questionnaire in 2009, six years on average post randomisation. This questionnaire includes demographic characteristics, self-reported history of angina, heart attack and stroke, self-reported smoking status, IPAQ, simple dietary behaviour questions, EuroQoL (EQ-5D), Short Form-8 (SF-8), and the adapted version of the Health Services Research Unit Aberdeen questionnaire for the use of medication and services.

### Costs of the intervention

The economic analysis will establish the NHS costs of the initial screening programme for type 2 diabetes from a patient and health service perspective. We will examine the cost-effectiveness of the multifactorial intensive treatment of patients with screen-detected type 2 diabetes from a health service perspective.

### Participant retention

The retention rate at one year follow-up was 85%. In order to maximise retention, we are reimbursing patients' travel at follow-up assessment. We have also been sending annual Christmas cards to all participants. A few months before the start of the five-year assessment, we will send a newsletter to all participants outlining the one-year results and plans for inviting them back for re-measurement.

### Participant safety

Screening equipment was enrolled in the HemoCue quality assurance programme. The glucose tolerance test was undertaken by trained staff in dedicated testing centres. Treatment algorithms have been developed with advice from local diabetes specialists who also contributed to the initial and follow-up practice-based training sessions for primary care staff involved in diabetes care. The responsibility for prescribing and management decisions remains with the general practitioners. Classes of medication are only recommended within licensed indications.

In Cambridge, an independent Trial Steering Committee meets regularly and makes recommendations on ethical or safety aspects. At the European level, a Data Monitoring and Ethics Committee receives periodic reports on deaths and hypoglycaemic episodes. Termination of the study would be determined on the basis of mortality. Based on general trials stopping rules, it was suggested that the first interim analysis blind to study group (using data from the three countries) be undertaken when the total number of deaths reaches 200. The rule for termination is a significant difference in mortality between the IT and RC groups at a level of significance of 0.001.

## Statistical procedures

### Analysis

#### (i) Effect of intensive multi-factorial treatment

Analysis will be by intention-to-treat allowing for clustering of patients by practice. This will be supported by sensitivity analyses, assuming a range of outcomes for non-completers informed by baseline data. The main analyses will compare outcomes between patients with screen-detected diabetes receiving routine care (RC) and those receiving intensive treatment (IT), adjusting for differences in baseline variables. The primary perspective for cost analysis will be the health service.

At one year comparisons will be made on modelled 10-year cardiovascular disease (CVD) risk [39] and on secondary outcomes including individual cardiovascular risk factors, health utility, functional health status, and costs. The costs of the intensive intervention will then be compared with unit change in health utility. At five-years, analyses will include comparisons of main outcomes (fatal and non-fatal macrovascular events) and secondary outcomes (microvascular events, individual cardiovascular risk factors, all-cause mortality, health utility, functional health status, and costs).

#### (ii) Population effects of screening

People at high risk of having undiagnosed diabetes in the screening practices (IT and RC) will be compared to those in the no screening (control) practices to assess the impact of screening on mortality, cardiovascular morbidity, health status, self-reported diet, physical activity and health service costs using ONS and questionnaire data. This will be done using an intention to screen analysis. For the mortality analysis the primary outcome will be all-cause mortality and the secondary outcomes cardiovascular, cancer and other causes of mortality. Mortality, cardiovascular morbidity, health status, diet, and physical activity among people at high risk of having undiagnosed diabetes will also be compared between IT and RC groups in an intention to treat analysis to quantify the potential wider benefits of the practice-based intensive treatment intervention package.

### Sample size

The sample size calculation was based on estimates of uptake and prevalence of undiagnosed diabetes from the Ely study between 1990 and 1992 [53]. IT vs. RC comparison of individual risk factors was originally based on 1000 screen-detected patients (500 in the IT and RC groups). Assuming 95% confidence and 80% power and an average practice list of 7,500 people, about 2,500 will be aged 40–69 years. Of these around 30% (750) will be at high risk of prevalent undiagnosed diabetes. Given a 70% uptake of screening [53] 525 would be tested and 60 would have prevalent undiagnosed diabetes per practice, of these 42 should complete one year follow-up [54]. The study design exhibits clustering of patients within practices. Typical values of intra-class correlations range from 0.01 to 0.1; we have previously reported correlations of 0.047 for HbA_1c _and 0.045 for BMI in people with diabetes one year after diagnosis [54]. For clusters of 42 patients the design effect is therefore 3 (range 1.4 to 5.0). Therefore using our previous diabetes cohort data [53,54], (1000 screen-detected cases would allow detection of the following clinically important differences between IT and RC groups: 0.5% in mean HbA_1c_, (difference between groups at one year in the UKPDS was 0.7% [55]), 11.5 mmHg systolic blood pressure, 1.5 kg/m^2 ^in body mass index, 10% in the proportion smoking, a 5 point difference in mean EuroQol health utility index [47] and 1.3 in mean anxiety level [49]. These estimations were initially completed for a total of 28 practices in the IT and RC arms. Given the lower than expected prevalence of diabetes within practices (<42 diabetic patients per practice), we recruited more practices, hence reducing the impact of clustering and improving the power of the study. 867 patients diagnosed with diabetes were finally enrolled.

Prior to the development and validation of a CVD risk score incorporating glycaemic control, the original sample size calculation was based on differences in individuals risk factors such as HbA_1c _and BMI. With the increased number of practices and smaller patients per practice, power was re-assessed using one-year follow-up data using risk factors making up the UKPDS ten-year modelled CVD risk (excluding the unavailable but rare component of atrial fibrillation). This was based on the initial 293 diabetic patients recruited to the RC arm of the study and accounted for clustering (intracluster correlation of 0.0185). It was estimated that there was 90% power at the 5% level of significance to detect a relative effect of 20% in the mean ten-year modelled CVD risk assuming one-year retention of 70% (600 patients in 48 practices).

## Discussion

*ADDITION-Cambridge *is designed to assess the feasibility and cost-effectiveness of a stepwise screening and intensive multi-factorial treatment programme for type 2 diabetes in a defined high-risk group accessible through primary care.

A targeted stepwise approach to screening is supported by the high proportion of undiagnosed diabetes in the UK [53], and the low performance of screening tests as stand alone assessments [56]. *ADDITION-Cambridge *assesses the feasibility of a combination of a diabetes risk score with various biochemical tests as a screening strategy in primary care. Although developed and tested in datasets from population-based surveys [21-23], the performance and yield of this risk score when used as part of a programme in an existing healthcare setting remain uncertain.

The treatment phase of this study has been designed to assess the costs and benefits of early multifactorial therapy in individuals with screen-detected diabetes with the ultimate aim of reducing the risk of cardiovascular events. Trials suggest that intensive treatment of people with type 2 diabetes is beneficial [8,57]. Much of the benefit of early intervention in screen-detected diabetes would depend upon the associated reduction of cardiovascular risk [6]. The treatment algorithm used in *ADDITION-Cambridge *is based on the Steno-2 regimen [8] which was tested in clinically diagnosed patients with diabetes at an advanced stage of the disease. The effectiveness of this regimen in people at an early stage of the disease has yet to be demonstrated. The patient education aspects of the early treatment programme have been informed by reviews on interventions to prevent weight gain [58], educational and psychosocial interventions for adults with diabetes [59], and trials of physical activity promotion [60]. These support the view that an education programme, especially one based on social, behavioural and psychological theory and evidence, can increase the effectiveness of behavioural change strategies [61,62].

*ADDITION-Cambridge *will provide evidence about the benefits, harms and costs of implementing a screening and early treatment programme for type 2 diabetes. The results will be of relevance to policy decisions about screening for diabetes, and subsequent management of people early in the course of the disease. Results will also inform approaches to health promotion, the management of chronic disease and risk, and will have implications for the training of practitioners in diabetes care.

## Competing interests

The authors declare that they have no competing interest.

## Authors' contributions

SJG, NJW and ALK are the principal investigators for the *ADDITION-Cambridge *trial. AT is the trial statistician, KMW and RSB are the trial co-ordinators, JBE, RKS and SJG drafted the manuscript. All authors read and approved the final manuscript. SJG is the paper guarantor

## The ADDITION- Cambridge team

Simon J. Griffin, Nicholas J. Wareham, Ann-Louise Kinmonth, Andrew T. Prevost Kate M. Williams, Roslyn S. Barling, Tom Fanshawe, Ryan Butler, Nicola Popplewell, Lincoln A. Sargeant, Paul Roberts, Matt Sims, Fiona Whittle, Julie Grant, James Brimicombe, Wendy Hardeman, Stephen Sutton, Ruhul Amin, Adam Dickinson, Justin B. Echouffo Tcheugui, Rebecca K. Simmons, Francis Finucane, Joana Mitchell, the Field, Data Management, IT and Study Coordination teams of the Medical Research Council Epidemiology Unit. The General Practice and Primary Care Research Unit at the University of Cambridge and the Medical Research Council Epidemiology Unit in Cambridge jointly coordinated the baseline and one-year follow-up phases of the study

## Independent Trial Steering Committee in Cambridge

Professors Nigel Stott (Chair), John Weinman, Richard Himsworth, and Paul Little.

## Data Monitoring and Ethics Committee

Per Winkel, Jørn Wetterslev and Christian Gluud from The Copenhagen Trial Unit (CTU), Centre for Clinical Intervention Research, Rigshospitalet, Copenhagen University Hospital.

## Pre-publication history

The pre-publication history for this paper can be accessed here:


